# Supplementary material for: Association between CHADS2, CHA2DS2-VASc, ATRIA, and Essen Stroke Risk Scores and Functional Outcomes in Acute Ischemic Stroke Patients Who Received Endovascular Thrombectomy
Source: J Clin Med. 2022 Sep 23;11(19):5599. doi: 10.3390/jcm11195599 (PMC9570925; doi:10.3390/jcm11195599)
Supplement: Supplementary file 1 [file jcm-11-05599-s001.zip › jcm-1863235-supplementary.pdf]

SUPPLEMENTAL MATERIAL

Association between $CHADS_2$, CHA_2DS_2-VASc, ATRIA, and Essen stroke risk scores and functional outcomes in acute ischemic stroke patients who received endovascular thrombectomy

Hyung Jun Kim, MD,[1] Moo-Seok Park, MD,[1] Joonsang Yoo, MD,[2] Young Dae Kim, MD, PhD,[3] Hyungjong Park, MD,[4] Byung Moon Kim, MD, PhD,[5] Oh Young Bang, MD, PhD,[6] Hyeon Chang Kim, MD, PhD,[7] Euna Han, PhD,[8] Dong Joon Kim, MD, PhD, [5] Joonnyung Heo, MD,[3] Jin Kyo Choi, MD,[9] Kyung-Yul Lee, MD,[10] Hye Sun Lee, PhD,[11] Dong Hoon Shin, MD, PhD,[12] Hye-Yeon Choi, MD, PhD,[13] Sung-Il Sohn, MD, PhD,[4] Jeong-Ho Hong, MD, PhD,[4] Jong Yun Lee, MD, PhD,[14] Jang-Hyun Baek, MD,[15] Gyu Sik Kim, MD,[16] Woo-Keun Seo, MD, PhD,[6] Jong-Won Chung, MD,[6] Seo Hyun Kim, MD, PhD,[17] Sang Won Han, MD,[18] Joong Hyun Park, MD,[18] Jinkwon Kim, MD, PhD,[3] Yo Han Jung, MD, PhD,[10] Han-Jin Cho, MD, PhD,[19] Seong Hwan Ahn, MD,[20] Sung Ik Lee, MD,[21] Kwon-Duk Seo, MD,[16] Yoonkyung Chang,[23] Hyo Suk Nam, MD, PhD,[3] and Tae-Jin Song, MD, PhD,[1] on behalf of the SECRET study investigators

Table S1. Univariate logistic regression analysis of the risk of an unfavorable outcome.

Variables	Unadjusted<br>OR (95% CI)	*p*-value
Age, per-1-year increase,	1.045 (1.027-1.062)	<0.001
Female sex	1.060 (0.716-1.569)	0.770
BMI, per-1-kg/m^2 increase	0.930 (0.885-0.977)	0.004
**Vascular risk factors**		
Hypertension	1.068 (0.681-1.677)	0.774
Diabetes mellitus	3.027 (2.011-4.555)	<0.001
Hypercholesterolemia	1.023 (0.691-1.516)	0.909
Current smoking	0.577 (0.342–0.982)	0.039
eGFR < 60 mL/min	2.640 (1.766-3.948)	<0.001
**Comorbidities**		
Atrial fibrillation	1.464 (0.987-2.170)	0.058
Heart failure	2.275 (1.092-4.740)	0.028
Coronary disease	0.613 (0.399-0.942)	0.026
Peripheral artery disease	1.833 (0.615-5.460)	0.277
Previous infarction	1.687 (1.045-2.724)	0.032
Previous hemorrhage	1.709 (0.667-4.786)	0.264
**Medication before admission**		
Prior antiplatelet therapy	1.252 (0.823-1.903)	0.294
Prior anticoagulant therapy	0.635 (0.374-1.077)	0.102

Prior statin therapy	1.075 (0.705-1.640)	0.736
Initial NIHSS score, per-1-score increase	1.172 (1.127-1.219)	<0.001
**Treatment**		
tPA		
-IA thrombolysis alone	Reference	
-Combined IV/IA thrombolysis*	0.566 (0.378-0.846)	0.006
Stent-retriever alone	0.501 (0.317-0.794)	0.003
Aspiration alone	3.434 (1.250-9.437)	0.017
Combined stent-retriever/ aspiration**	0.638 (0.389-1.075)	0.104
Number of stent-retriever passes, per-1-passes increase	1.224 (1.092-1.372)	0.001
Onset to puncture, per-1-min increase	1.000 (1.000-1.001)	0.793
LNT-to-puncture time (within 6hrs)	1.341 (0.567-1.745)	0.542
**Stroke etiology**		
Cardioembolic	reference	
Large artery atherosclerosis	0.688 (0.394-1.202)	0.189
Undetermined or others	0.940 (0.598-1.479)	0.790
**Image finding after EVT**		
mTICI 2b-3	0.135 (0.067-0.272)	<0.001
Hemorrhagic transformation	8.942 (4.308-18.558)	<0.001
**Stroke risk score**		
CHA_2DS_2VASc score	1.386 (1.213-1.583)	<0.001
$CHADS_2$ score	1.731 (1.409-2.127)	<0.001
ATRIA score	1.162 (1.096-1.233)	<0.001

Essen score	1.289 (1.111-1.495)	0.001

OR, odd ratio; CI, confidence interval; BMI, body mass index; eGFR, estimated glomerular filtration rate; National Institutes of Health Stroke Scale, NIHSS; tPA, tissue plasminogen activator; IA, intra-arterial; IV, intravenous; LNT, last normal time; EVT, endovascular thrombectomy; mTICI, modified thrombolysis in cerebral infarction

* administration of tissue plasminogen activator prior to endovascular thrombectomy

**cases in which stent retriever and aspiration were performed simultaneously or sequentially

Table S2. Multivariate analysis for stroke risk score correlated with the unfavorable outcome among 404 patients with EVT (Model 2)

	**$CHADS_2$**		**CHA_2DS_2VASc**		**ATRIA**		**Essen**	
Variables	OR (95% CI)	*p*-value	OR (95% CI)	*p*-value	OR (95% CI)	*p*-value	OR (95% CI)	*p*-value
BMI, per 1-kg/m^2 increase	0.947 (0.896-1.002)	0.060	0.978 (0.921-1.038)	0.457	0.974 (0.918-1.034)	0.386	0.953 (0.899-1.010)	0.106
Current smoking	0.947 (0.504-1.781)	0.867	0.969 (0.515-1.826)	0.923	0.951 (0.503-1.800)	0.878	0.692 (0.364-1.315)	0.261
eGFR < 60 mL/min	1.472 (0.884-2.451)	0.138	1.709 (1.055-2.767)	0.029	1.657 (0.997-2.753)	0.051	1.523 (0.919-2.522)	0.103
Atrial fibrillation	0.826 (0.504-1.356)	0.451	0.855 (0.523-1.398)	0.532	0.886 (0.544-1.443)	0.627	0.834 (0.509-1.367)	0.472
Heart failure	1.134 (0.453-2.842)	0.788	1.266 (0.492-3.255)	0.625	1.781 (0.755-4.204)	0.187	1.874 (0.798-4.397)	0.149
Coronary disease	0.394 (0.236-0.656)	<0.001	0.414 (0.250-0.687)	<0.001	0.426 (0.258-0.706)	<0.001	0.304 (0.170-0.545)	<0.001
Previous infarction	1.416 (0.802-2.499)	0.230	1.427 (0.812-2.509)	0.217	1.167 (0.606-2.250)	0.644	1.415 (0.802-2.496)	0.231
Initial NIHSS score, per 1-score increase	1.165 (1.118-1.214)	<0.001	1.161 (1.115-1.210)	<0.001	1.160 (1.114-1.209)	<0.001	1.162 (1.115-1.210)	<0.001
**Risk scoring score**								
Per-1-point increase	1.678 (1.327-2.121)	**<0.001**	1.257 (1.073-1.471)	**0.005**	1.091 (1.013-1.176)	**0.021**	1.350 (1.096-1.664)	**0.005**

OR, odd ratio; CI, confidence interval; BMI, body mass index; eGFR, estimated using the glomerular filtration rate; National Institutes of Health Stroke Scale, NIHSS

Table S3. Multivariate analysis for stroke risk score correlated with the unfavorable outcome among successful recanalization patients (Model 1)

	$CHADS_2$		CHA_2DS_2VASc		ATRIA		Essen	
Variables	OR (95% CI)	*p*-value	OR (95% CI)	*p*-value	OR (95% CI)	*p*-value	OR (95% CI)	*p*-value
BMI, per-1-kg/m^2 increase	0.994 (0.885-1.116)	0.913	1.017 (0.910-1.136)	0.766	1.023 (0.913-1.146)	0.695	1.011 (0.902-1.131)	0.857
eGFR < 60 mL/min	1.327 (0.517-3.406)	0.557	1.526 (0.610-3.820)	0.367	1.315 (0.505-3.426)	0.575	1.633 (0.647-4.121)	0.299
Heart failure	0.718 (0.145-3.546)	0.684	0.840 (0.161-4.376)	0.836	1.301 (0.321-5.278)	0.713	1.668 (0.432-6.432)	0.458
Initial NIHSS score, per 1-score increase	1.212 (1.112-1.320)	<0.001	1.213 (1.113-1.322)	<0.001	1.120 (1.101-1.308)	<0.001	1.218 (1.118-1.327)	<0.001
**IV thrombolysis**								
IA thrombolysis alone	Reference		Reference		Reference		Reference	
Combined IA/IV thrombolysis*	0.222 (0.093-0.532)	<0.001	0.213 (0.089-0.510)	<0.001	0.207 (0.086-0.497)	<0.001	0.195 (0.082-0.463)	<0.001
**IA thrombolysis**								
Stent-retriever alone	0.709 (0.218-2.304)	0.567	0.708 (0.217-2.309)	0.567	0.689 (0.212-2.244)	0.536	0.670 (0.207-2.168)	0.504
Aspiration alone	1.545 (0.578-2.516)	0.478	1.347 (0.375-2.248)	0.589	1.257 (0.853-2.221)	0.322	1.321 (0.322-2.325)	0.477
Number of stent-retriever passes, per-1-passes increase	1.302 (0.972-1.743)	0.077	1.299 (0.968-1.744)	0.086	1.328 (0.980-1.800)	0.067	1.241 (0.939-1.639)	0.130
**Imaging finding after EVT**								
mTICI 2b-3	0.144 (0.036-0.575)	0.006	0.132 (0.033-0.523)	0.004	0.130 (0.033-0.507)	0.003	0.119 (0.031-0.450)	0.002

Hemorrhagic transformation	90.381 (9.213-886.623)	<0.001	89.740 (9.540-844.195)	<0.001	99.500 (10.553-938.187)	<0.001	79.941 (9.017-708.712)	<0.001
**Risk scoring score**								
Per-1-point increase	1.728 (1.084-2.754)	0.022	1.297 (0.979-1.718)	0.070	1.161 (1.000-1.348)	0.049	1.089 (0.750-1.583)	0.654

OR, odd ratio; CI, confidence interval; BMI, body mass index; eGFR, estimated using the glomerular filtration rate; National Institutes of Health Stroke Scale, NIHSS; IV, intravenous; IA, intra-arterial; EVT, endovascular thrombectomy; mTICI, modified thrombolysis in cerebral infarction

* administration of tissue plasminogen activator prior to endovascular thrombectomy

Table S4. Clinical and imaging characteristics according to the degree of clinical outcome after EVT (AF-related stroke and non-AF-related stroke)

	**AF-related stroke – followed up to 3 months (N=190)**			**Non-AF-related stroke – followed up to 3 months (N=214)**		
	**Favorable outcome (mRS 0-2) (N=81)**	**Unfavorable outcome (mRS 3-6) (N=109)**	***P*-value**	**Favorable outcome (mRS 0-2) (N=110)**	**Unfavorable outcome (mRS 3-6) (n=104)**	***P*-value**
Age, years, mean (SD)	76.6 ± 10.1	81.9 ± 10.0	<0.001	70.7 ± 14.4	78.3 ± 14.1	<0.001
Female, (%)	47 (58.0%)	52 (47.7%)	0.207	39 (35.5%)	47 (45.2%)	0.189
BMI (kg/m^2)	20.3 ± 4.1	20.2 ± 4.1	0.866	22.1 ± 3.7	20.1 ± 4.4	<0.001
**Vascular risk factors**						
Hypertension, (%)	60 (74.1%)	91 (83.5%)	0.159	82 (74.6%)	70 (67.3%)	0.310
Diabetes mellitus, (%)	38 (46.9%)	73 (67.0%)	0.009	44 (40.0%)	75 (72.1%)	<0.001
Hypercholesterolemia, (%)	39 (48.2%)	47 (43.1%)	0.588	46 (41.8%)	49 (47.1%)	0.521
Current smoking, (%)	11 (13.6%)	9 (8.3%)	0.346	30 (27.3%)	20 (19.2%)	0.220
eGFR <60 mL/min, (%)	39 (48.20%)	73 (67.0%)	0.014	33 (30.0%)	58 (55.8%)	<0.001
**Comorbidities**						
Heart failure, (%)	6 (7.4%)	20 (18.4%)	0.050	5 (4.6%)	6 (5.8%)	0.924
Coronary disease, (%)	31 (38.3%)	31 (28.4%)	0.203	36 (32.7%)	22 (21.2%)	0.080
Peripheral artery disease, (%)	2 (2.5%)	4 (3.7%)	0.961	3 (2.7%)	6 (5.8%)	0.443
Previous infarction, (%)	20 (24.7%)	36 (33.0%)	0.278	14 (12.7%)	21 (20.2%)	0.197
Previous hemorrhage	2 (2.5%)	6 (5.5%)	0.506	5 (4.6%)	7 (6.7%)	0.561

**Medication before admission**						
Prior antiplatelet therapy, (%)	29 (35.8%)	40 (36.7%)	>0.999	28 (25.5%)	34 (32.7%)	0.310
Prior anticoagulation therapy, (%)	27 (33.3%)	26 (23.9%)	0.202	11 (10.0%)	3 (2.9%)	0.068
Prior statin therapy, (%)	31 (38.3%)	33 (30.3%)	0.318	27 (24.6%)	35 (33.7%)	0.188
Initial NIHSS score, median ((IQR)	13 (9-16)	18 (14-21)	<0.001	11 (7-15)	18 (12.75-21.25)	<0.001
**Treatment**						
IA thrombectomy alone, (%)	36 (44.4%)	76 (69.7%)	<0.001	64 (58.2%)	69 (66.4%)	0.276
Combined IV/IA thrombolysis*, (%)	45 (55.6%)	33 (30.3%)	<0.001	86 (47.5%)	52 (34.4%)	0.022
Stent-retriever alone, (%)	70 (86.4%)	72 (66.1%)	0.003	84 (76.4%)	72 (69.2%)	0.308
Aspiration alone, (%)	0 (0.0%)	11 (10.1%)	0.009	5 (4.6%)	7 (6.7%)	0.691
Combined stent-retriever/aspiration**, (%)	11 (13.6%)	26 (23.9%)	0.113	21 (19.1%)	25 (24.0%)	0.475
Number of stent-retrieval passes (SD)	1.8 ± 1.1	2.5 ± 2.4	0.010	1.8 ± 1.4	2.4 ± 2.3	0.013
Onset to puncture, min, mean (SD)	312.2 ± 305.4	318.6 ± 278.7	0.883	365.8 ± 528.6	353.0 ± 323.1	0.830
LNT-to-puncture time (within 6hrs)	63 (77.8%)	78 (71.6%)	0.423	59 (56.7%)	38 (51.4%)	0.577
**Image finding after EVT**						
mTICI 2b-3	77 (95.1%)	78 (71.6%)	<0.001	104 (94.6%)	73 (70.2%)	<0.001
Hemorrhagic transformation	24 (29.6%)	70 (64.2%)	<0.001	76 (69.1%)	57 (54.8%)	0.044
**Pre-admission stroke risk score, median (IQR)**						
$CHADS_2$ score	4 (3-4)	4 (3-5)	<0.001	2 (1-2)	2 (1-3)	0.001
CHA_2DS_2VASc score	2 (1-3)	3 (2-3)	<0.001	3 (2-4)	4 (2-4)	<0.001
ATRIA score	8 (6-9)	9 (8-10)	<0.001	6 (1-8)	8 (2-10)	<0.001

Essen score	3 (3-4)	4 (3-4)	0.008	3 (2-4)	3.5 (2-4)	0.050

EVT, endovascular thrombectomy; AF, atrial fibrillation; mRS, modified Rankin Scale; SD, standard deviation; BMI, body mass index; eGFR, estimated glomerular filtration rate; NIHSS, National Institutes of Health Stroke Scale; IQR, interquartile range; IA, intra-arterial; IV, intravenous; LNT, last normal time

* administration of tissue plasminogen activator prior to endovascular thrombectomy

**cases in which stent retriever and aspiration were performed simultaneously or sequentially

Table S5. Multivariate analysis for stroke risk score correlated with the unfavorable outcome in patients with AF-related stroke (Model 1)

	$CHADS_2$		CHA_2DS_2VASc		ATRIA		Essen	
Variables	OR (95% CI)	*p*-value	OR (95% CI)	*p*-value	OR (95% CI)	*p*-value	OR (95% CI)	*p*-value
BMI, per-1-kg/m^2 increase	1.014 (0.909-1.130)	0.809	1.037 (0.935-1.150)	0.498	1.041 (0.937-1.156)	0.454	1.029 (0.925-1.144)	0.602
eGFR < 60 mL/min	1.442 (0.589-3.529)	0.423	1.675 (0.702-3.995)	0.245	1.522 (0.607-3.816)	0.370	1.721 (0.714-4.149)	0.226
Heart failure	0.912 (0.209-3.981)	0.902	1.247 (0.277-5.607)	0.773	1.768 (0.491-6.357)	0.383	1.992 (0.572-6.941)	0.279
Initial NIHSS score, per 1-score increase	1.153 (1.071-1.242)	<0.001	1.157 (1.074-1.245)	<0.001	1.150 (1.068-1.238)	<0.001	1.164 (1.082-1.252)	<0.001
**IV thrombolysis**								
IA thrombolysis alone	Reference		Reference		Reference		Reference	
Combined IA/IV thrombolysis*	0.298 (0.135-0.655)	0.003	0.289 (0.132-0.630)	0.002	0.288 (0.132-0.630)	0.002	0.268 (0.124-0.579)	<0.001
**IA thrombolysis**								
Stent-retriever alone	0.668 (0.226-1.972)	0.465	0.666 (0.227-1.957)	0.459	0.648 (0.222-1.896)	0.429	0.650 (0.222-1.905)	0.432
Aspiration alone	1.214 (0.578-2.516)	0.418	1.317 (0.345-2.148)	0.529	1.258 (0.553-2.121)	0.522	1.325 (0.222-2.525)	0.479
Number of stent-retriever passes, per-1-passes increase	1.290 (0.990-1.681)	0.059	1.273 (0.984-1.648)	0.066	1.301 (0.998-1.695)	0.052	1.239 (0.963-1.595)	0.096

**Imaging finding after EVT**								
mTICI 2b-3	0.123 (0.031-0.491)	0.003	0.115 (0.029-0.450)	0.002	0.115 (0.030-0.438)	0.002	0.112 (0.030-0.414)	0.001
Hemorrhagic transformation	4.170 (1.904-9.131)	<0.001	4.250 (1.961-9.211)	<0.001	4.412 (2.037-9.588)	<0.001	4.311 (2.009-9.250)	0.001
**Risk scoring score**								
Per-1-point increase	1.766 (1.135-2.749)	0.012	1.268 (0.976-1.649)	0.076	1.114 (0.979-1.348)	0.103	1.102 (0.782-1.552)	0.580

OR, odd ratio; CI, confidence interval; BMI, body mass index; eGFR, estimated using the glomerular filtration rate; National Institutes of Health Stroke Scale, NIHSS; IV, intravenous; IA, intra-arterial; EVT, endovascular thrombectomy; mTICI, modified thrombolysis in cerebral infarction

* administration of tissue plasminogen activator prior to endovascular thrombectomy

Table S6. Comparison of area under curve (AUC) of each stroke risk score by two. (Univariate ROC analysis)

AUC1 vs. AUC2	**AUC1**	**AUC2**	***p*-value**
$CHADS_2$ score vs. CHA_2DS_2VASc score	0.654	0.644	0.406
CHA_2DS_2VASc score vs. ATRIA score	0.644	0.663	0.375
ATRIA score vs. Essen score	0.663	0.596	0.014
$CHADS_2$ score vs. ATRIA score	0.654	0.663	0.699
$CHADS_2$ score vs. Essen score	0.654	0.596	<0.001
CHA_2DS_2VASc score vs. Essen score	0.644	0.596	0.011

AUC, area under curve; ROC, receiver operating characteristic

Table S7. Comparison of area under curve (AUC) of each stroke risk score by two in successful recanalization patients. (Univariate ROC analysis).

AUC1 vs. AUC2	**AUC1**	**AUC2**	***p*-value**
$CHADS_2$ score vs. CHA_2DS_2VASc score	0.621	0.613	0.567
CHA_2DS_2VASc score vs. Atria score	0.613	0.642	0.204
ATRIA score vs. Essen score	0.642	0.570	0.014
$CHADS_2$ score vs. ATRIA score	0.621	0.642	0.389
$CHADS_2$ score vs. Essen score	0.621	0.570	0.004
CHA_2DS_2VASc score vs. Essen score	0.613	0.570	0.050

AUC, area under curve; ROC, receiver operating characteristic

Table S8. ROC curve analysis (AUC), NRI, and IDI of predictive models for unfavorable outcome in EVT patients

	Model*	Model + $CHADS_2$	Model + CHA_2DS_2VASc	Model + ATRIA	Model + Essen	*p***	*p*†	*p*‡	*p*§
ROC curve (AUC)	0.877 (0.844 to 0.910)	0.880 (0.847 to 0.912)	0.877 (0.844 to 0.910)	0.878 (0.845 to 0.911)	0.878 (0.845 to 0.911)	0.430	0.905	0.647	0.662
Category-based NRI (95% CI)		3.6 (-0.7 to 8.0)	1.5 (-1.8 to 4.7)	14.3 (7.5 to 21.1)	3.6 (0 to 7.1)	0.103	0.381	<0.001	0.051
Continuous-based NRI (95% CI)		38.8 (19.8 to 57.8)	19.1 (-0.3 to 38.4)	54.4 (35.6 to 73.1)	28.3 (9.3 to 47.3)	<0.001	0.053	<0.001	0.004
Relative IDI (95% CI)		0.6 (-0.1 to 1.4)	0.1 (-0.3 to 0,5)	3 (1.2 to 4.8)	0.3 (-0.2 to 0.9)	0.111	0.507	0.001	0.194

ROC, receiver-operating characteristics; AUC, area under curve; NRI, net reclassification improvement; IDI, integrated discrimination improvement; EVT, endovascular thrombectomy; CI, confidence interval

*Model included BMI, coronary disease, initial NIHSS score, tPA use, stent-retriever alone, aspiration alone, and number of stent-retriever passes.

**Comparison between Model and Model + $CHADS_2$

† Comparison between Model and Model + CHA_2DS_2VASc

‡ Comparison between Model and Model + ATRIA

§ Comparison between Model and Model + Essen
